# Dental implants significantly increase adjacent tooth loss risk due to root fracture

**DOI:** 10.1038/s41432-024-01052-0

**Published:** 2024-08-17

**Authors:** Kelvin I. Afrashtehfar, Jad Moriss Kazma, Islam Yahia, Aesa A. Jaber

**Affiliations:** 1 Department of Reconstructive Dentistry and Gerodontology, School of Dental Medicine, University of Bern, Bern, Switzerland; 2 College of Dentistry, Ajman University, Ajman, UAE; 3 Private Practice, Abu Dhabi, UAE; 4 MOH Internship Clinics, College of Dentistry, Ajman University, Ajman, UAE

**Keywords:** Dental implants, Occlusion

## Abstract

**Design:**

This retrospective cohort study aimed to investigate the risk and variables of tooth loss for teeth adjacent to dental implants compared to teeth nonadjacent to implants. The study followed the STROBE guidelines and was approved by the Institutional Review Board.

**Cohort selection:**

The study included patients treated with dental implants at UCSF School of Dentistry between 2000 and 2020. The inclusion criteria for teeth adjacent to implants required the implant to support a fixed prosthesis and a follow-up period of at least 12 months. Nonadjacent teeth also required a follow-up period of at least 12 months. Teeth were excluded if they had a hopeless prognosis or were planned for extraction before the completion of restorative treatment.

**Data analysis:**

Data were extracted from electronic health records, including patient demographics, dental histories, and outcomes for teeth adjacent and nonadjacent to implants. Statistical analyses, including Kaplan-Meier survival plots, log-rank tests, and multivariate logistic regression, were used to compare tooth survival and identify aetiologies of tooth loss.

**Results:**

The study included 787 patients, with 2048 teeth adjacent and 15,637 teeth nonadjacent to implants. The 10-year cumulative survival rate was 89.2% for teeth adjacent to implants and 99.3% for nonadjacent teeth. Teeth adjacent to implants had a significantly higher risk of tooth loss (Odds Ratio [OR] 13.15). The primary etiology of tooth loss adjacent to implants was root fracture (45.2%), followed by caries (28.9%), periodontitis (24.1%), and endodontic failure (1.8%). For nonadjacent teeth, periodontitis was the leading cause of tooth loss (51.9%).

**Conclusions:**

The study found that teeth adjacent to dental implants had a significantly higher risk of tooth loss, primarily due to root fractures. The findings suggest that dental implants may act as an iatrogenic factor, increasing the risk of complications for adjacent teeth. Conservative management of natural dentition should be prioritized, with emphasis on stringent periodontal surveillance and effective home care. Future research should focus on prospective studies to further explore these associations and improve clinical outcomes.

## A Commentary on

**Chen H H, Lin G H, Kao R T, Yeh Y T**.

Survival rate of teeth adjacent and nonadjacent to dental implants: A retrospective cohort study. *J Periodontol* 2024; 10.1002/JPER.23-0739.

**GRADE Rating:** 

## Commentary

Dental implants provide sustainable long-term function and esthetics, making them a popular choice for replacing missing teeth, with 10-year survival and success rates above 90%^1,2^. Despite their high survival rates, complications such as caries, fractures, and hypermobility often affect teeth adjacent to implants^3^. The absence of proper proximal contact can increase the risk of caries and periodontal disease in adjacent teeth^4^. Additionally, the size, shape, and emergence profile of implant-supported restorations impact proximal embrasure morphology, which can affect the health of adjacent teeth^5^. Recent studies exposed the need for further investigation to mitigate these iatrogenic risks^6^. Dental implants are more susceptible to crestal bone loss under eccentrical occlusal forces, requiring occlusal load management strategies that may inadvertently overload adjacent teeth, increasing the risk of cracks or fractures^7,8^. Given the limited evidence on how dental implants influence the outcomes of adjacent teeth, the reviewed retrospective cohort study by Chen et al.^9^ aims to explore the risk and variables of tooth loss for teeth adjacent to dental implants compared to nonadjacent teeth, crucial for optimizing treatment outcomes and preserving oral health.

The retrospective cohort study stands out due to its robust sample size of 787 patients and an average follow-up period of almost 5 years. This extensive data collection improves the reliability of the findings. Additionally, the differentiation between teeth adjacent and nonadjacent to dental implants allows for precise comparison, while the use of Kaplan-Meier survival analysis and multivariate logistic regression provides a detailed and statistically sound examination of risk factors for tooth loss.

Additionally, the study’s meticulous approach in categorizing aetiologies of tooth loss and the clear statistical representation of the survival rates add depth to the findings. The detailed dental history of adjacent teeth, including restorations, root canal treatments, and periodontal therapy, provide comprehensive data into contributing factors. The study’s adherence to rigorous reporting standards, such as the STROBE guidelines, further supports its scientific rigor.

Despite its strengths, the study presents several limitations. The retrospective nature of the cohort study, relying on electronic health records, means causal relationships cannot be firmly established. Confounding factors, such as parafunctional habits and variations in oral hygiene, could potentially skew results. Additionally, the absence of detailed periodontal prognosis for adjacent teeth and the exclusion of dental history for nonadjacent teeth may limit the comprehensiveness of the conclusions drawn.

Future research should focus on conducting well-designed randomized controlled trials with extended follow-up periods to provide more definitive evidence on the risk factors associated with tooth loss adjacent to dental implants. Standardizing methodologies and outcomes will enable more reliable comparisons. Exploring the biomechanical interactions between implant occlusion and adjacent teeth^10^, particularly in the context of periodontal health, could discover valuable information for reducing the complications in teeth next to implants.

To sum up, Chen et al. effectively demonstrated a significantly higher risk of tooth loss for teeth adjacent to dental implants, with root fracture identified as the primary etiology. These findings encourage clinicians to realize the need for careful planning and management of implant-supported restorations to mitigate adverse effects on adjacent natural teeth.

Practice points

- Clinicians should closely monitor the health of teeth adjacent to dental implants, recognizing the higher risk of complications such as root fractures and caries.
- When planning implant placements, clinicians must consider the potential risks to adjacent teeth and incorporate preventive measures to mitigate these risks.
